# Exploring the multifaceted antitumor activity of axitinib in lung carcinoids

**DOI:** 10.3389/fendo.2024.1433707

**Published:** 2024-07-10

**Authors:** Monica Oldani, Maria Celeste Cantone, Germano Gaudenzi, Silvia Carra, Alessandra Dicitore, Davide Saronni, Maria Orietta Borghi, Angela Lombardi, Michele Caraglia, Luca Persani, Giovanni Vitale

**Affiliations:** ^1^ Laboratory of Geriatric and Oncologic Neuroendocrinology Research, IRCCS, Istituto Auxologico Italiano, Milan, Italy; ^2^ Laboratory of Endocrine and Metabolic Research, IRCCS, Istituto Auxologico Italiano, Milan, Italy; ^3^ Department of Medical Biotechnology and Translational Medicine, University of Milan, Milan, Italy; ^4^ PhD Program in Experimental Medicine, University of Milan, Milan, Italy; ^5^ Department of Clinical Sciences and Community Health, University of Milan, Milan, Italy; ^6^ Experimental Laboratory of Immuno-Rheumatology, Istituto Auxologico Italiano, IRCCS, Milan, Italy; ^7^ Department of Precision Medicine, University of Campania “L. Vanvitelli”, Naples, Italy; ^8^ Laboratory of Molecular and Precision Oncology, Biogem Scarl, Ariano Irpino, Italy

**Keywords:** lung carcinoid, tyrosine kinase inhibitors, axitinib, cell cycle, senescence, mitotic catastrophe, reactive oxygen species

## Abstract

**Introduction:**

Lung carcinoids (LCs) are a type of neuroendocrine tumor (NET) that originate in the bronchopulmonary tract. LCs account for 20–25% of all NETs and approximately 1–2% of lung cancers. Given the highly vascularized nature of NETs and their tendency to overexpress vascular growth factor receptors (VEGFR), inhibiting angiogenesis appears as a potential therapeutic target in slowing down tumor growth and spread. This study evaluated the long-term antitumor activity and related mechanisms of axitinib (AXI), a VEGFR-targeting drug, in LC cell lines.

**Methods:**

Three LC cell lines (NCI-H727, UMC-11 and NCI-H835) were incubated with their respective EC_50_ AXI concentrations for 6 days. At the end of the incubation, FACS experiments and Western blot analyses were performed to examine changes in the cell cycle and the activation of apoptosis. Microscopy analyses were added to describe the mechanisms of senescence and mitotic catastrophe when present.

**Results:**

The primary effect of AXI on LC cell lines is to arrest tumor growth through an indirect DNA damage. Notably, AXI triggers this response in diverse manners among the cell lines, such as inducing senescence or mitotic catastrophe. The drug seems to lose its efficacy when the DNA damage is mitigated, as observed in NCI-H835 cells.

**Conclusion:**

The ability of AXI to affect cell viability and proliferation in LC tumor cells highlights its potential as a therapeutic agent. The role of DNA damage and the consequent activation of senescence seem to be a prerequisite for AXI to exert its function.

## 1 Introduction

Lung carcinoids (LCs) are a type of neuroendocrine tumor (NET) that originate in the bronchopulmonary tract. LCs account for 20–25% of all NETs and approximately 1–2% of lung cancers. More than 80% of LCs are diagnosed at TNM stage I or II (1). In advanced tumors, the goals of therapeutic management are to control tumor proliferation and manage functioning syndromes through a multidisciplinary approach (2–4). However, LCs can be highly heterogeneous, responding differently to treatments. This variability can make it challenging to define the therapeutic approach and to find new effective therapies (5).

NETs are highly vascularized tumors, with 64–80% of cases exhibiting an overexpression of endothelial vascular growth factor (VEGF) and Vascular Endothelial Growth Factor receptors 1, 2 and 3 (VEGFR-1, -2, and -3) (6–8). Based on this, the inhibition of angiogenesis could have a key role in reducing the metastatic potential of these neoplasms. Several investigations have reported an important involvement of angiogenesis in the progression of lung NETs. Angiogenic factors, such as VEGF, Angiopoietin 2 (ANG2) and prokineticin 2 (PROK2) correlate with tumor aggressiveness (9–12). Uncontrolled activity of angiogenic factors in lung NETs can contribute to invasive tumor behavior, endothelial cell growth, and occurrence of metastasis (13). The significant roles played by VEGF in the growth and spread of lung NETs are supported by higher serum VEGF levels detected in patients with larger primary tumor sizes, nodal involvement, and distant metastases (13). Notably, LCs exhibit higher expression levels of VEGFR-2 and -3 compared to the other lung NETs (8). Moreover, a significant increase in VEGF expression is strongly associated with reduced survival in patients with LCs (14). All these findings have prompted to consider monoclonal antibodies against VEGF and VEGFR tyrosine kinase inhibitors (TKIs) as a possible treatment for lung NETs (9, 10, 15–21). Among these drugs, axitinib (AXI), a selective tyrosine kinase inhibitor targeting VEGF receptors (22–24), has shown to improve outcomes in patients with NETs (17).

In our previous research (25), we evaluated the antitumor activity of AXI on different human LC cell lines. We demonstrated that AXI reduced *in vitro* the viability rate of LC cell lines and induced a cell cycle arrest in the G_2_/M phase after 3 days of treatment. AXI inhibited tumor-induced angiogenesis and reduced the invasiveness of LC cells in zebrafish *Tg(fli1a: EGFP)^y1^* embryos. All these findings supported the potential of AXI as a therapeutic agent in LCs. However, observing the effects on cells after a short-term treatment period, we cannot exclude the possibility that LC cells may develop drug resistance with prolonged treatment.

In the present study, we evaluated the long-term antitumor activity of AXI on human LC cell lines in inducing programmed cell death programs (senescence, apoptosis and mitotic catastrophe) and/or cell cycle arrest. Recognizing these cellular outcomes is of paramount importance to understand the cell behavior changes induced by AXI and to design new therapeutic approaches.

## 2 Materials and methods

### 2.1 Cells and reagents

Human LC cell lines NCI-H727, UMC-11 and NCI-H835 were purchased from ATCC and standard protocols were followed for their maintenance. These three different cell lines are representative of well-differentiated pulmonary NETs, classified as typical LCs. Among these three cell lines, the responses to pharmacological treatments can vary significantly, reflecting the heterogeneity reported in this tumor and making them useful for comparative studies (26–28). In brief, cells were routinely seeded in T75 flasks containing RPMI medium (EuroClone™, Milan, Italy) and supplemented with 10% heat-activated fetal bovine serum (FBS) (EuroClone™, Milan, Italy) and 10^5^ U·L−1 penicillin/streptomycin (EuroClone™, Milan, Italy). Prior to experiments, LC cell lines were counted using a standard hemocytometer. Cells utilized in all experiments were below 5 passages. Axitinib (AXI) (MedChemExpress, Monmouth Junction, NJ, USA) was diluted in dimethyl sulfoxide (DMSO) at the concentration of 10^-2^ M and stored at -80 C°.

### 2.2 Cell cycle and apoptosis evaluation

Cell cycle and apoptosis were investigated after 6 days of incubation with AXI. 1 x 10^5^ cells/well were seeded in 6-well plates in duplicate for both NCI-H727 and UMC-11, while 3 x 10^5^ cells/well were counted for NCI-H835. Twenty-four hours after seeding, the cell medium was replaced with RPMI supplemented with 0.1% DMSO (referred to as CTR) or with the specific EC_50_ dose of AXI for each cell line. In detail, 2 x 10^-6^ M of AXI was used to treat NCI-H727 cell line, while a concentration of 4 x 10^-7^ M and 2.4 x 10^-7^ M were tested on UMC-11 and NCI-H835 cell lines, respectively. After 3 days, the RPMI medium was replaced again preserving the above-mentioned conditions for the CTR and AXI treatment. The sixth day, cells were harvested by trypsinization, washed with PBS, and collected with centrifugation. For cell cycle, a propidium iodide (PI) solution (50 μg/ml PI, 0.05% Triton X-100 and 0.6 μg/ml RNase A in 0.1% sodium citrate, all from Sigma-Aldrich® Merck KGaA, Darmstadt, Germany) was added to stain the pellets at 4°C for 30 minutes in the dark. For apoptosis, each sample was resuspended in 100 μl of 1X binding buffer (BB: 1.4M NaCl, 0.1M HEPES/NaOH, pH 7.4, 25 mM CaCl2) and incubated with 5 μl Annexin-V-fluorescein isothiocyanate (FITC) (BD Pharmingen, San Diego, CA, USA) and 10 μl PI (50 μg/ml in PBS) for 15 minutes at room temperature in the dark. Additional 400 μl of 1X BB have been added to each sample before the acquisition. Both Samples stained for cell cycle and apoptosis were analyzed through BD FACSLyric™ (BD Pharmingen, San Diego, CA, USA) flow cytometer using BD FACSuite™ Software on 10,000 events (BD Pharmingen, San Diego, CA, USA) (29).

### 2.3 Cell lysis and western blot analysis

LC cells were plated as described in the previous paragraph and incubated without (CTR) and with AXI for 6 days. Thereafter, the seeded cells were scraped in 50 µl of radio-immuno-precipitation assay lysis buffer (RIPA: 50 mM Tris-HCl pH 7.5, 150 mM NaCl, 1% NP-40, 0.5% sodium deoxycholate, 0.1% SDS) added with phosphatase (Roche, Basel, Switzerland) or protease inhibitors cocktail tablets (Sigma-Aldrich® Merck KGaA, Darmstadt, Germany). The cellular lysates were harvested by centrifuging at 15,000g for 30 minutes at 4°C. PierceTM BCA Protein Assay Kit (Thermo ScientificTM, Pierce Biotechnology, Illinois, USA) was used following manufacturer’s instructions to detect the protein content in each supernatant. Ten micrograms of proteins per lane were separated on Mini-PROTEAN TGX 4–20% precast polyacrylamide gels (Bio-Rad, Bio-Rad Laboratories, Inc, USA) and transferred through iBlot Gel Transfer Stacks Nitrocellulose (Invitrogen by Thermo Fisher Scientific, Waltham, MA, USA). Subsequently, nitrocellulose membranes were incubated with specific first antibody at 4°C overnight. Antibodies, all diluted 1:1000 and provided by Cell Signaling Technology (Danvers, MA, USA), were as follow: Caspase-3 (D3R6Y) Rabbit Ab; Poly(ADP-ribose)polymerase (PARP) (46D11) Rabbit Ab; Phospho-Chk1(Ser345) (133D3) Rabbit mAb; Chk1 (2G1D5) Mouse mAb; p21 WAF1/Cip1 (12D1) Rabbit mAb; Phospho-p53 (Ser15) (16G8) Mouse mAb; Cyclin-B1 Rabbit Ab; Phospho-Histone H2A.X (Ser139) (D7T2V) Mouse mAb (γ-H2AX); Nuclear factor erythroid 2-related factor 2 (Nrf2) (D1Z9C) XP Rabbit mAb; Kelch-like ECH-associated protein 1 (Keap1) (D6B12) Rabbit mAb; and βActin (8H10D10) mouse mAb. After incubation with anti-mouse or anti-rabbit IgG, HRP-linked Ab (dilution 1:5000) (Cell Signaling Technology, Danvers, MA, USA), signals were detected through ECL Star Enhanced Chemiluminescent Substrate (Euroclone, Milan, Italy), and band exposition were revealed by Azure Imaging Systems (Azure Biosystems, Dublin, CA, USA). Band intensities were expressed as absolute unit, normalized by the level of β-actin expression in each sample and, at the end, compared with the untreated cell band.

### 2.4 Analysis of reactive oxygen species

The general production of intracellular reactive oxygen species (ROS) and nitric oxide (•NO) was revealed by the oxidation of 2′,7′-dichlorofluorescin diacetate (H2DCFDA) (Sigma Chemical Co., St. Louis, MO). Human LC cell lines were seeded in 96-well plates at density of 6 x 10^3^ cells/well for NCI-H727 and UMC-11 and 5 x 10^4^ cells/well for NCI-H835. The day after, cell medium was replaced without or with different concentration of AXI, as reported above. At the end of the third or sixth day, cells were incubated in the dark with 5 μM H2DCFDA diluted in PBS. At the end of 20 minutes incubation at 37°C, the probe fluorescence intensity was measured by the FL-1 channel (excitation = 485 nm; emission = 528 nm) using a microtiter plate reader (VICTOR X3, PerkinElmer) and analyzed by the PerkinElmer 2030 Manager software for Windows. H2DCFDA fluorescence was normalized by the total protein content (PierceTM BCA Protein Assay Kit, see above) in each sample.

### 2.5 β-gal senescence assay

Cells were seeded in 6-well plates in duplicate and incubated without and with AXI for 6 days, as previously described. Thereafter, we performed the β-galactosidase staining (pH 6.0) for measuring cell senescence, according to manufacturer’s instructions (Cell Signaling Technology, Danvers, MA, USA). All images of stained cells were acquired by the inverted and epifluorescent Leica DMIRE2 microscope (Leica Microsystems, Illinois, USA) using the same parameters of magnification and light exposure. The blue pixel percentage in the total area of each image were performed using (Fiji Is Just) ImageJ software. At least 3 images were analyzed for each condition.

### 2.6 Morphological analysis

After 6 days of AXI treatment, LC cells were stained with two fluorescent dyes to quantify the crucial morphological parameters related to the mitotic catastrophe phenomenon. Hoechst Stain (Invitrogen™) diluted 1:1000 in PBS was used to identify cell nuclei, whereas Cell Tracker Green CMFDA (5-chloromethylfluorescein diacetate) staining (Invitrogen™) was used to monitoring the whole cell body. Human LC cell lines were seeded in duplicate in 6-well plates and incubated without and with AXI for 6 days, as described so far. At this point, two similar procedures have been applied for adherent (NCI-H727 and UMC-11) or in suspension (NCI-H835) cell lines. The NCI-H727 and UMC-11 cells were first incubated with Hoechst stain and then with Cell Tracker Green (0.125 mM). For both passages, an incubation time of 10 minutes at 37°C in the dark and an intermediate and gentle wash with PBS was required. On the other hand, for enhancing the adherence of cells to the plate, NCI-H835 cell line was primarily transferred into a new 6-well plate previously poly-lysinated and, after 3h of incubation, cells were stained with both Hoechst, as previously described. Detection of cell fluorescence for Hoechst blue images (excitation = 350 nm; emission = 450 nm filter) was performed through the inverted and epifluorescent Leica DMIRE2 microscope (Leica Microsystems, Illinois, USA). Thereafter, blue fluorescent signals for each cell in the images were quantified using (Fiji) Imagej software for analyzing the morphological parameters such as Area, Perimeter, and Circularity. All the quantified images have maintained the same pixel size and at least 3 images were analyzed for each condition.

### 2.7 Statistical analysis

All experiments were performed at least in triplicate. Statistical differences among groups were first calculated applying a Normality test (Shapiro-Wilk test), after we have carried out either an unpaired t test or a Two-ways ANOVA test followed by a *post hoc* test (Sidak’s multiple comparison test). A p value <0.05 was considered significant. The values reported in the figures represent the mean ± Standard Error of the Mean (SEM). For statistical analysis, GraphPad Prism 8.0.1 (GraphPad Software, San Diego, CA, USA) was used.

## 3 Results

### 3.1 Human LC cell lines do not activate apoptosis after 6 days of AXI treatment

**Figure 1:**
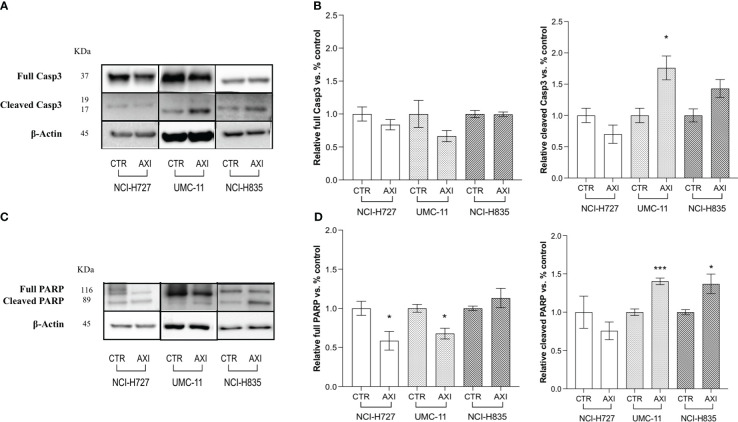
Examination of apoptosis activation by Western blot analysis after 6 days of incubation with or without AXI in each LC cell line. **(A, C)** are representative Western blot images for each target in LC cell lines. Histograms **(B, D)** summarize the relative expression change of each target. The targets analyzed are full and cleaved Caspase-3 **(A, B)** and PARP **(C, D)**. β-Actin is used as a loading control. Values represent the mean ± S.E.M. of a minimum of 3 independent experiments. The significance is calculated by performing an unpaired t-test between the control (CTR) and the treated group: *p<0.05; ***p<0.001.

**Figure 2:**
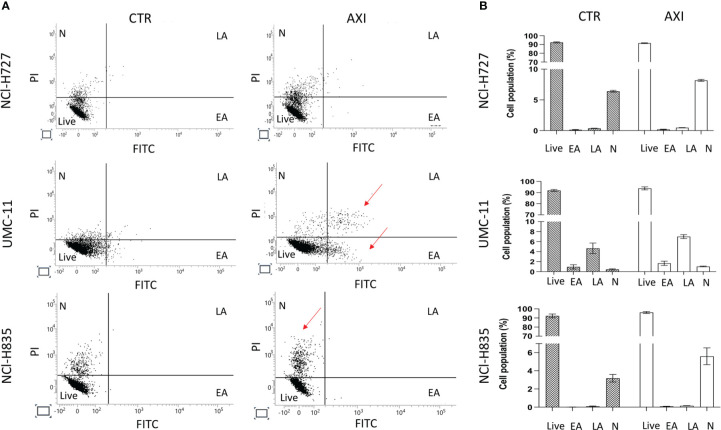
Apoptosis Analyses. **(A)** shows a representative distribution of the cell population in untreated cells (CTR) and AXI treated samples. A single event, represented as a dot, is correlated to FITC/PI fluorescence detection. The dials in each graph indicate the percentage of live cells (Live), cells in the early stages of apoptosis (EA), cells in the late stages of apoptosis (LA) or necrosis (N). The red arrows indicate where LC cells have been typically accumulated after treatment with AXI. Histograms in **(B)** summarize the average percentage ± S.E.M. of Live, EA, LA and N of three independent experiments. The results are analyzed by comparing the value in each dial between AXI and CTR samples (2way ANOVA test).

We have previously reported that the three LC cell lines showed different responsiveness to AXI in terms of inhibition of cell viability (25). UMC-11 cell line was the most sensitive to AXI treatment, with a maximal growth inhibitory effect of approximately 93%. The maximal growth inhibition induced by AXI on NCI-H727 was about 65%. Interestingly, NCI-H835 cells were sensitive to lower concentrations of AXI if compared to NCI-H727 cell line. However, the growth inhibitory effects of AXI on NCI-H727 were more pronounced at higher concentrations (**Table 1**). The AXI maximal growth inhibitory effect generally increased over the time (after 3 and 6 days of treatment) for all LC cell lines (**Table 1**). Based on these observations, we analyzed the possible activation of apoptosis after 6 days of treatment, which could be due to the decrease in viability observed in all tumor cell lines by either MTT or MTS assays (25). Therefore, each cell line was treated with its own EC_50_ at 6 days of incubation (**Table 1**). Western blot analyses revealed that apoptotic mechanisms were activated in both UMC-11 and NCI-H835 cells, as demonstrated by a significant increase in cleaved caspase-3 (UMC-11 cells) and PARP (both cell lines) after treatment with AXI (**Figure 1**). However, cytofluorimetric analyses after Annexin-V and PI labelling showed that the percentage of dead LC cells were extremely low and did not increase after treatment with AXI (**Figure 2**). Moreover, a slight accumulation of cells in early and late apoptosis (EA and LA) or necrosis (N) was observed in UMC-11 and NCI-H835 cell lines, respectively (**Figure 2**).

**Table 1:** Summary of MTT or MTS data.

Cell lines	EC_50_ 3D (M)	EC_50_ 6D (M)	Maximal inhibition effect 3D	Maximal inhibition effect 6D
NCI-H727	8,6*10^-6^	1,9*10^-6^	-27%	-65%
UMC-11	5*10^-6^	4*10^-7^	-63%	-93%
NCI-H835	2.8*10^-7^	2*10^-7^	-20%	-47%

The table presents EC50 absolute values and the maximal inhibition effect obtained from three independent experiments assessing the effect of AXI on cell viability after 3 (3D) or 6 days (6D) of AXI treatment [data derived from 25]. These values are calculated by generating dose-response curves following MTT assays (for NCI-H727 and UMC-11) or MTS assays (for NCI-H835).

### 3.2 Human LC cell lines undergo cell cycle arrest after 6 days of AXI treatment

**Figure 3:**
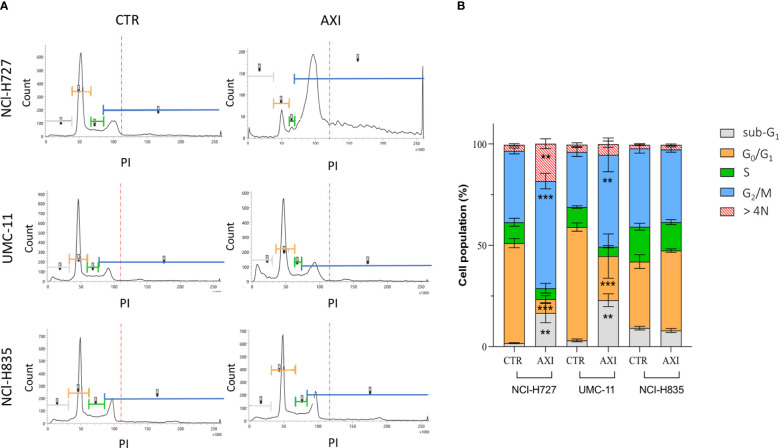
Modulation of cell cycle in NCI-H727, UMC-11 and NCI-H835 cell lines after 6 days of incubation with AXI. The different phases of the cell cycle are associated with the same reference colors in both images. **(A)** Compendium of representative profiles of the cell cycle distribution of the different cell lines without or with AXI. **(B)** Graph of the mean percentage of cells in the sub-G_1_, G_0_/G_1_, S, G_2_/M phases and >4N for control (CTR) and treated samples. The results of three independent experiments are analyzed by comparing the cell percentage in each phase between AXI and CTR samples (2way ANOVA test): ** p<0.01; ***p<0.001.

Flow cytometric analysis after labelling of methanol-fixed cells with PI showed that the cell cycle was not altered before or after AXI in NCI-H835 (**Figure 3**). However, NCI-H727 and UMC-11 cell lines underwent to cell cycle arrest in G_2_/M phase after treatment with AXI (**Figure 3**). Specifically, a significant reduction of the percentage of both cell lines in the G_0_/G_1_ phase and an increase in the G_2_/M phase were observed after AXI (**Figure 3**). Cell cycle arrest at this stage could be indicative of not repairable DNA damage. Moreover, the observed increase of the cell population in the sub-G_1_ phase, typically indicative of DNA fragmentation without detectable cell death, could be another indication of a possible DNA damage. AXI significantly induced an increase of the percentage of polyploid (>4N) cells only in NCl-H727 cells (**Figure 3**).

### 3.3 AXI induces DNA damage in human LC cell lines

**Figure 4:**
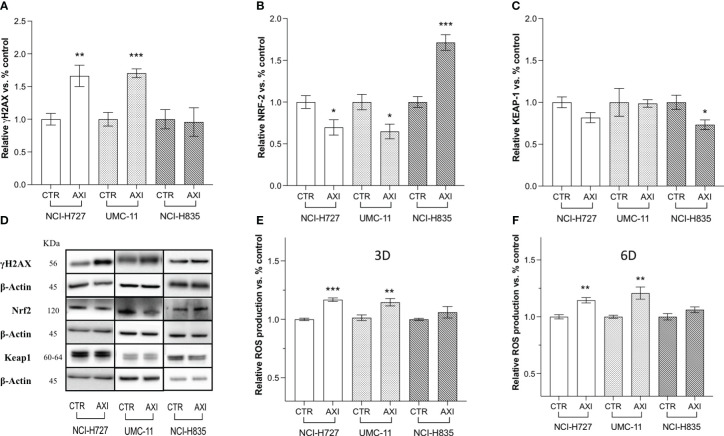
Western blot analysis of DNA damage and quantification of ROS in untreated (CTR) and AXI-treated cells. Histograms **(A–C)** summarize the relative expression change of one target after 6 days of treatment with AXI in each LC cell line. **(D)** Representative Western blot images for each target in LC cell lines. The analyzed targets are phospho-Histone H2A.X (Ser139) (γH2AX), Nrf2, Keap1. βActin is used as loading control. Panels **(E, F)** show the relative quantification of intracellular ROS production after 3 and 6 days of incubation, respectively. Values represent the mean ± S.E.M. of a minimum of three independent experiments. Significance is calculated by performing an unpaired t-test between the control and treated groups: *p<0.05; ** p<0.01; ***p<0.001.

The next step was to determine whether AXI could actually induce DNA damage in LC cells. In this view, γ-H2AX, a variant of the H2A histone family, was analyzed by Western blot, as it is rapidly phosphorylated at sites of DNA double-strand breaks and serves as a marker for these lesions. γ-H2AX levels were significantly enhanced after 6 days of AXI treatment in NCI-H727 and UMC-11, while no changes were recorded in NCI-H835 cells (**Figures 4A, D**). As suggested by Morelli et al. (30), AXI appeared to cause DNA damage through increased intracellular ROS. Indeed, we found a significant increase in ROS production after AXI treatment, measured at both short (3 days) and long times (6 days) of exposure to AXI in both NCI-H727 and UMC-11 cells (**Figures 4E, F**). As ROS levels remained high over time in NCI-H727 and UMC-11cells (**Figures 4E, F**), it is possible that ROS induced DNA damage and this condition might be sufficient to maintain a cell cycle arrest in both cell lines. On the other hand, the NCI-H835 cell line maintained a stable level of ROS over time (**Figures 4E, F**), with a concurrent downregulation of Keap1 (p<0.05) and upregulation of Nrf2 (p< 0.001) after incubation with AXI (**Figures 4B–D**).

### 3.4 Pathways activated to counteract DNA damage in NCI-H727 and UMC-11 cell lines

**Figure 5:**
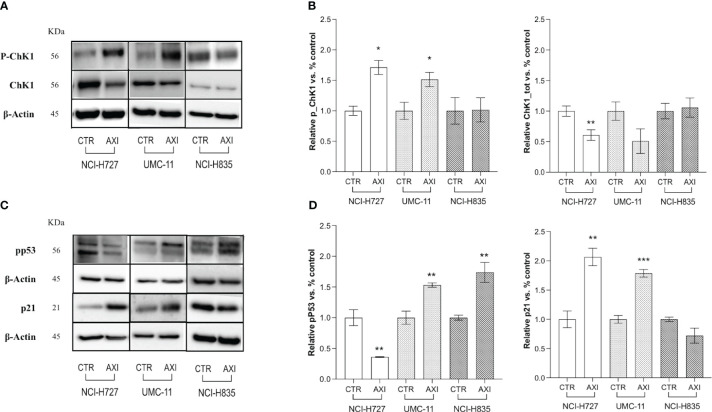
Examination of cell cycle arrest by Western blot analysis. **(A, C)** Representative Western blot images for each target in LC cell lines. Histograms **(B, D)** summarize the change in expression of one target after 6 days of treatment with AXI in each LC cell line. The analyzed targets are phospho-Chk1(Ser345) (P-ChK1), total Chk1 (Chk1) **(A, B)**, phospho-p53 (Ser15) (pp53) and p21 Waf1/Cip1 **(C, D)**. βActin is used as a loading control. Values represent the mean ± S.E.M. of a minimum of three independent experiments. The significance is calculated by performing an unpaired t-test between the control and the treated group: *p<0.05; ** p<0.01; ***p<0.001.

Two critical pathways related to DNA damage and cell cycle arrest in the G_2_/M phase were analyzed by Western blot: Chk1 and p53. In particular, during DNA damage or replication stress, ChK1 is activated by phosphorylation at Ser 345 (P-ChK1). Once activated, ChK1 affects cell cycle progression leading to cell accumulation, mainly at the G_2_/M phase, as previously shown. We recorded an activation of ChK1 with an increase of about 1.5-fold in both NCI-H727 and UMC-11 cells after treatment with AXI (**Figures 5A, B**). The phosphorylation of p53 at Ser15 (pp53) is another regulatory event in cell response to DNA damage, contributing to both p53 stabilization and activation. In fact, after activation, pp53 induces cell cycle arrest at G_2_/M phase and/or apoptosis in case of irreparable DNA damage. The phosphorylation of p53 at Ser 15 was different in these two cell lines. In UMC-11 cells, pp53 was 1.5-fold increased if compared to untreated controls, whereas in NCI-H727 cells it was more 0.5-fold decreased after exposure to AXI (**Figures 5C, D**). Notably, the p21 (WAF1/Cip1) protein, one of p53 main downstream effectors and key regulator of the G_2_/M transition, was also augmented in UMC-11 cells after treatment with AXI (**Figures 5C, D**). However, a significative increase in p21 expression was also observed in NCI-H727 cells, apparently independent from the p53 activation (**Figures 5C, D**).

### 3.5 NCl-H727 and UMC-11 cell lines show senescence features after AXI treatment

**Figure 6:**
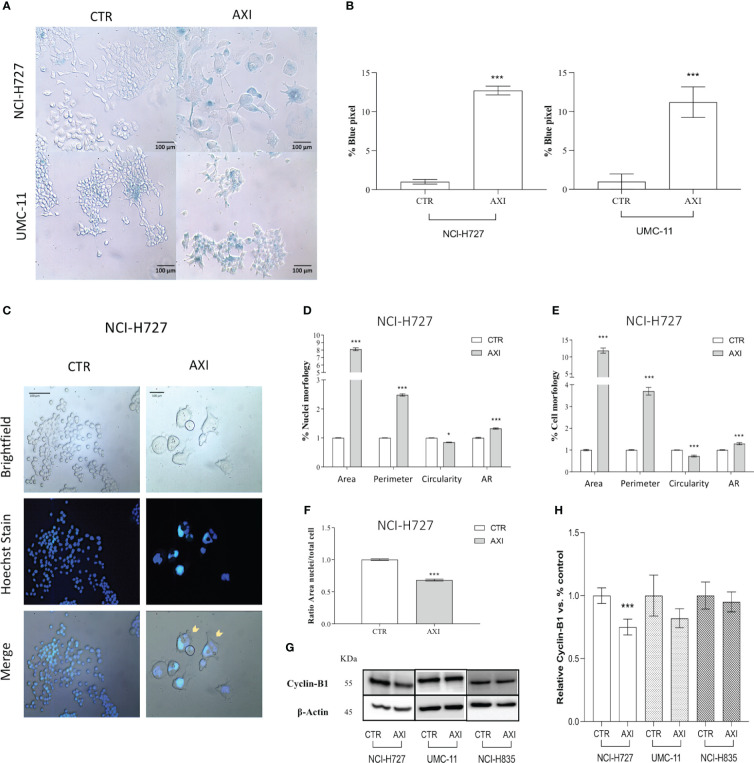
Evaluation of cell senescence and morphological changes of LC cells after treatment with AXI. **(A)** Representative bright field images of AXI-treated human LC and control cells (CTR) after staining with SA-β-gal. All quantified images have the same pixel size. **(B)** Histograms show the quantification of the blue pixel percentage present in the total image area of treated and control cells after staining with SA-β-gal. Values show the mean ± S.E.M. of a minimum of 3 independent experiments. In **(C)** LC cells are stained with a fluorescent dyes to quantify the main morphological parameters related to the mitotic catastrophe phenomenon. Hoechst dye is used to identify nuclei. **(D–F)** are representative of NCI-H727 cell line. Morphological parameters related to the nucleus or cell shape and their changes can be visualized in **(D, E)**, respectively. The analysis on the ratio between the cell area and nucleus are shown in **(F)**. Representative Western blot images and relative quantifications for the expression of Cyclin-B1 after 6 days of treatment with AXI in each LC cell line are shown in **(G, H)**. βActin is used as loading control. Values represent the mean ± S.E.M. of a minimum of three independent experiments. Significance was calculated by performing an unpaired t-test between the control and treated groups: *p<0.05; ***p<0.001.

We hypothesized that p21 high expression can also serve as a biomarker for the activation of senescence. Therefore, we evaluated the activity of senescence-associated β-galactosidase (SA-β-gal), a conventional hallmark of senescence. A significant (p<0.001) increase in SA-β-gal activity was recorded in both NCI-H727 and UMC-11 cell lines treated with AXI confirming the senescence induction (**Figures 6A, B**). On the other hand, NCI-H835 cell line did not exhibit any feature of senescence after treatment with AXI (data not shown). However, the distinct morphological alterations seen exclusively in NCI-H727 cells treated with AXI, namely their increased size and flattened shape, could be not only attributed to senescence occurrence but also to mitotic catastrophe.

### 3.6 AXI induces mitotic catastrophe in NCI-H727 cells

Tetraploid tumor cells observed in NCI-H727 (see section 3.2, >4N), intrinsically susceptible to mitotic aberrations, could be relevantly sensitive to the induction of mitotic catastrophe. Since mitotic catastrophe is characterized by large cells with multiple micronuclei, the shape of tumor cells and their nuclei was evaluated in LC cells treated with AXI. After staining with Hoechst 33258, only NCI-H727 cells had a significant enlargement of the nucleus for both their area and circumference after AXI (**Figures 6C, D**). As a result of abnormal mitosis, the nuclei lose their circularity, showing a more complex shape, as indicated by an increase in the aspect ratio (AR) value (**Figure 6D**). NCI-H727 cells area and perimeter also increased (**Figure 6E**). Moreover, after closer inspection of the nuclear to cytoplasmic area ratio, it was observed that despite an overall increase in cell size after treatment with AXI, the nuclei of NCI-H727 cells did not enlarge with a similar extent to occupy a larger part of the cell (**Figure 6F**). In addition, Western blot analysis indicated that polyploid and senescent NCI-H727 cells showed lower levels of cyclin-B1, additionally suggesting the potential role of cell cycle arrest and senescence in this context (**Figures 6G, H**).

## 4 Discussion

LCs are complex tumors that demand a multidisciplinary approach and a sophisticated therapeutic strategy (2–4). Recently, several TKIs have been investigated in LCs (31–36). Among these, AXI, a potent and selective second-generation inhibitor of VEGFRs, has demonstrated effectiveness in treating metastatic NETs (37), advanced hepatocellular carcinoma (HCC) (38), non-small cell lung cancer (23, 39), advanced renal carcinoma (40), epithelial ovarian cancer (EOC) (41). In a recent study (25), we demonstrated that AXI reduced cell viability in preclinical models of human LC cell lines. Specifically, in NCI-H727 AXI induced high expression of cleaved PARP and caspase-3. At the same time, AXI showed a potent anti-proliferative effect in lung NCI-H727 and UMC-11 cell lines that was correlated to cell cycle arrest in G_2_/M phase. Additionally, using *Tg(fli1a:EGFP)^y1^* zebrafish embryos implanted with the same LC cell lines, AXI was found to significantly inhibit tumor-induced angiogenesis and tumor cell invasiveness.

**Figure 7:**
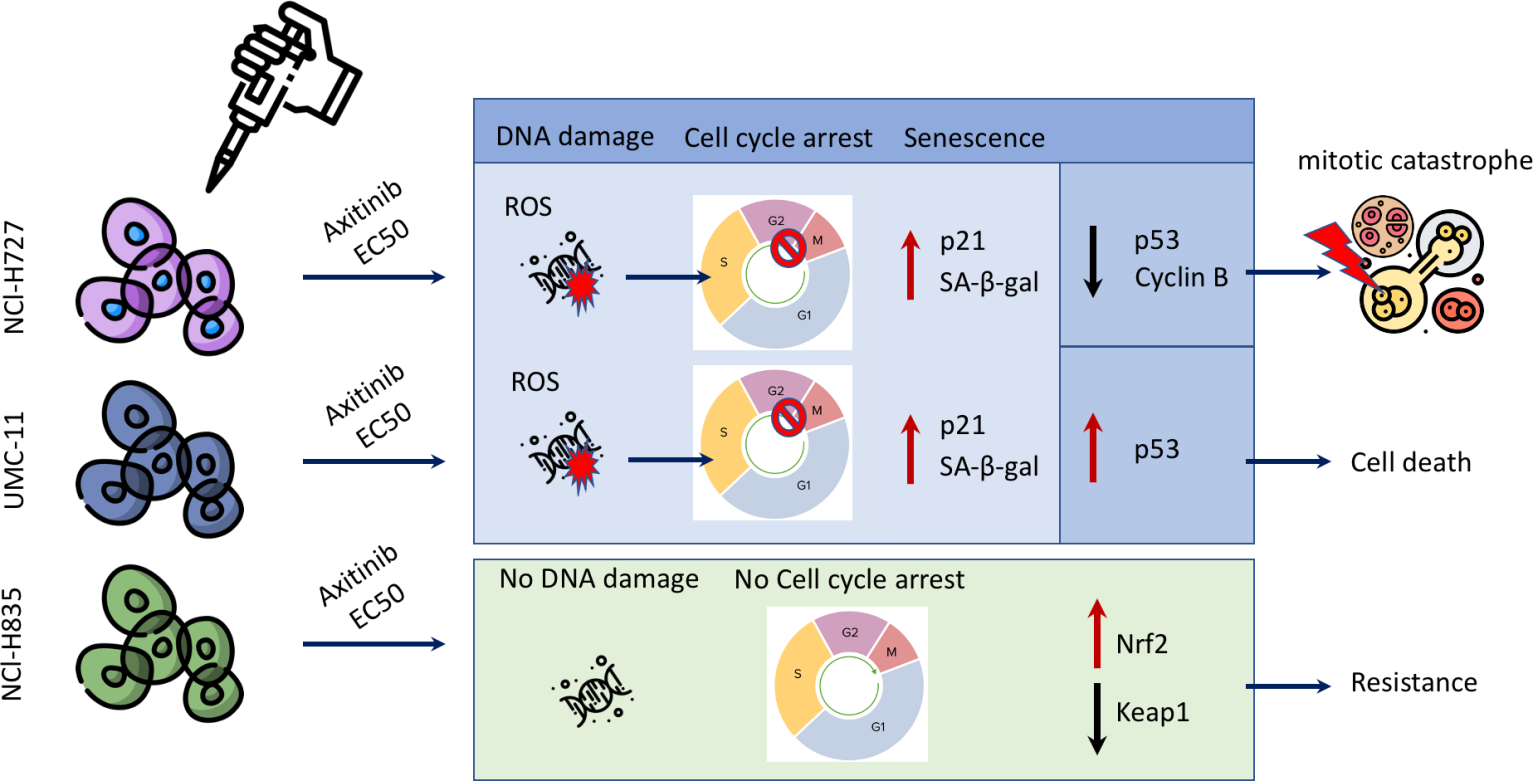
Scheme summarizing the different cell fates following treatment with AXI in LC cell lines.

Our current research has unveiled a range of anti-tumor effects exerted by AXI after prolonged treatment, as depicted in **Figure 7**. AXI inhibited cell viability in human LC cell lines in a time-dependent manner. By comparing the MTT or MTS results acquired after 3 or 6 days of cell exposure to AXI, we observed that the anti-tumor effect of this drug increased over time (**Table 1**). Notably, for the NCI-H727 cells, extending the treatment from 3 to 6 days resulted in a 4-fold reduction in the EC_50_ value and a 3-fold increase in the maximal growth inhibitory effect (**Table 1**). The UMC-11, the most sensitive cell line, exhibited a decrease in the EC_50_ value from 5*10^-6^ M to 4*10^-7^ M, with only around 10% of cells surviving at the maximum AXI concentration (**Table 1**). Even the relatively resistant NCI-H835 cell line showed a decrease in cell viability with the drug. Although the change in EC_50_ values between 3 and 6 days was not significant (from 2.8*10^-7^ M to 2*10^-7^ M), the drug reduced cell viability at the maximum inhibition dose by half (**Table 1**). The reduction in cell viability can be attributed either to the activation of cell death processes or to a cell cycle arrest. While our expectations were that prolonged exposure to AXI might increase cell mortality, the results revealed significant variability of these cells in the response to the drug (**Figure 7**).

NCI-H727 cells, which initiated apoptotic processes at a short incubation time (25), did not sustain the activation of the apoptotic pathways after 6 days. UMC-11 cell lines maintained an active apoptotic response, at least at molecular level. Finally, NCI-H835 cells, which did not display an early response to the drug during the short exposure (25), showed an increase in cleaved PARP that could be due to the potential activation of the apoptotic pathway. However, cytofluorimetry analyses indicated that the induction of cell death was not the primary mechanism behind the decrease in the cell viability after 6 days of treatment with AXI in all LC cell lines. Indeed, the number of cells that were annexin-V and/or PI positive after 6 days was generally low.

One potential drug tolerance strategy adopted by tumor cells is the modification of their own cell cycle (42). On this light, NCI-H835 cell line showed no cell cycle arrest, whereas both NCI-H727 and UMC-11 cell lines showed cell cycle arrest in the G_2_/M phase.

The underlying causes of this cell cycle arrest could be linked to the occurrence of DNA damage in the treated cells, as indicated by the notable elevation in γ-H2AX expression compared to the control cells observed in the Western blot analysis (43). Since the production of ROS can result in permanent DNA damage (44), we also assessed whether AXI could induce ROS increase in the cytosol of treated cells. We observed an upregulation of activated γ-H2AX and an increase in ROS levels only in NCI-H727 and UMC-11 cells, suggesting that AXI utilizes this mechanism to induce a block of cell cycle progression in the G_2_/M phase (**Figure 7**). Supporting this hypothesis, similar findings were reported in other *in vitro* studies on renal cell carcinoma (30, 45) and glioma cell lines (46) treated with AXI. DNA damage normally leads to apoptosis, but efficient DNA repair mechanisms prevent cell death, allowing cells to survive despite the initial damage. Furthermore, the rise in cell population in the sub-G_1_ phase in both NCI-H727 and UMC-11 cell lines, typically indicative of DNA fragmentation, without detectable cell death, suggests effective repair mechanisms or potential involvement of alternative cellular processes, such as senescence. These processes may represent protective responses to oxidative stress. Briefly, we hypothesize that all these results can indicate the presence of a potential reciprocal relationship between DNA damage response and ROS generation, which is adequate to sustain cell cycle arrest and, potentially, to trigger senescence in both cell lines (47). These data also suggest that the arrest of cell proliferation and cellular senescence induced by DNA damage could play a crucial role in the response of tumors to AXI, similar to what occurs in chemotherapy (48–50). Indeed, we evaluated the expression of pp53, Chk1, activated by the presence of DNA damage, and of p21 (WAF1/Cip1), playing a major role in cell cycle arrest and senescence, respectively (51–54). Both UMC-11 and NCI-H727 cell lines showed high levels of p21 and activation of Chk1 after AXI. However, the activation of p53 was observed only in UMC-11 cells. Moreover, accordingly to the role of the activation of both p53 and p21 in the growth arrest of senescent cells, UMC-11 cells showed positive staining for senescence-specific SA-β-gal (55, 56). This may help to explain why, after 6 days, a substantial number of apoptotic cells were not detected, but the activation of cell death pathways remained evident. In UMC-11 cells, pp53 seems to regulate the cell cycle by activating p21. This protein stops cell proliferation to allow DNA repair by entering the cells in a state of senescence. However, since the DNA repair seems to be ineffective or impossible due to severe damage, pathways that lead to cell death are activated. In contrast, NCI-H727 cell line, which did not show p53 phosphorylation, underwent to senescence and mitotic catastrophe occurring through a p53-independent pathway (57) (**Figure 7**). In NCI-H727 cells, where p53 is reported to be defective, the cell cycle arrest is ineffective, leading to the accumulation of DNA damage and the inability to undergo complete mitosis. This scenario results in the increase of tetraploid cells, as shown after cytofluorimetric analyses. Interestingly, there are three different pathways associated to mitotic catastrophe, and only two lead to cell death (58–60). The first pathway, referred to as ‘mitotic death’ is characterized by increased levels of cyclin B1, which our cells do not appear to show. In the second pathway, cells exit mitosis through a process known as ‘slippage’ and undergo cell death in the subsequent G_1_ phase of the cell cycle (61, 62). This mechanism appears unlikely to explain the effects of AXI. The third pathway does not lead to cell death but instead induces senescence characterized by decreased levels of cyclin B1 (63, 64), as observed in our experimental model. Thus, in our study, the decline in cyclin B1 levels seems to play a role in polyploidization by sustaining an irreversible stop in the cell cycle. This prevents the proliferation of genomically unstable cells and potentially enables DNA replication in NCI-H727 cells undergoing AXI-induced senescence (49, 60, 65).

A separate consideration is necessary for the NCI-H835 cell line. The NCI-H835 cell line, which demonstrated no evidence of DNA damage and maintained a stable level of ROS over time, was able to activate a ROS resistance mechanism, probably through the Keap1/Nrf2 signaling. This mechanism, previously reported in renal cell carcinoma treated with AXI (45, 66), involves reduced Keap1 expression, increased Nrf2 expression, and overall decreased susceptibility to AXI. The Keap1/Nrf2 pathway is critical for antioxidant responses and cellular defense mechanisms, contributing significantly to tumor progression and resistance to chemotherapy and radiotherapy in different cancer types (67, 68). This mechanism occurring through increased Nrf2 activity was initially described in non-small cell lung cancer cells (69, 70), but was quickly also associated to high-grade pulmonary NETs (71). Moreover, Keap1 promoter hypermethylation was identified in about 50% of the tissues from patients with LCs (69). Looking at a possible correlation between methylation, mutations, and loss of heterozygosity (LOH) in the KEAP1 gene and the disease course, it was observed that the degree of KEAP1 inhibition showed a trend of association with a higher risk of tumor progression. By activating the Keap1/Nrf2 signaling pathway, NCI-H835 cells effectively mitigated the production of ROS and the consequent DNA damage that AXI could potentially induce, as observed in other tumor cell lines (45, 72). However, the specific reasons why only this particular tumor cell line activated this defense mechanism remain unclear, requiring further investigation.

A limitation of this study is the exclusive use of cell lines from LCs, due to the challenges of obtaining primary cultures from this tumor type, given the limited tissue availability and low mitotic activity.

In conclusion, AXI exhibits a time-dependent enhancement of efficacy in LC cell lines and, notwithstanding the diverse responses of cells, the role of DNA damage and the consequent activation of senescence following treatment seem to be a prerequisite for AXI to exert its antitumor activity. When cells manage to repair DNA damage, the effectiveness of the drug is reduced. However, it is evident that the variability of AXI in mechanisms of action could potentially represent an advantage in light of the heterogeneity reported in LCs. Nevertheless, AXI capacity to induce a range of anti-tumor effects, from apoptosis to senescence, and its significant impact on cell viability and proliferation suggest its role as a possible therapeutic agent. The inclusion of AXI in a poly-therapeutic approach could also improve the overall treatment efficacy, especially when considering the drug ability to induce cell cycle arrest. Given the complexity of tumor biology, additional studies are warranted to define the potential role of AXI in patients with advanced LCs.

## Data availability statement

The datasets presented in this study can be found in online repositories. The datasets for this study can be found in Zenodo (https://doi.org/10.5281/zenodo.11105090).

## Ethics statement

Ethical approval was not required in accordance with the local legislation and institutional requirements because only commercially available established cell lines were used.

## Author contributions

MO: Data curation, Formal analysis, Investigation, Validation, Visualization, Writing – original draft. MCC: Conceptualization, Data curation, Investigation, Validation, Visualization, Writing – review & editing. GG: Conceptualization, Writing – review & editing. SC: Conceptualization, Writing – review & editing. AD: Conceptualization, Data curation, Investigation, Writing – review & editing. DS: Data curation, Investigation, Writing – review & editing. MB: Data curation, Investigation, Validation, Writing – review & editing. AL: Writing – review & editing. MC: Writing – review & editing. LP: Writing – review & editing. GV: Resources, Supervision, Writing – review & editing.
